# Evidence and gap maps

**DOI:** 10.1002/cl2.1075

**Published:** 2020-03-11

**Authors:** Ashrita Saran

**Affiliations:** ^1^ Evidence Synthesis Specialist Campbell Collaboration

This issue of Campbell Systematic Reviews includes the first two evidence and gap maps (EGMs) to be published by the Campbell Collaboration. The first is a map of 396 studies of the impact of agroforestry on agricultural productivity, ecosystem services and human well‐being (Miller et al., 2019), copublished with the International Initiative for Impact Evaluation (3ie). The other EGM is of 166 studies on the effectiveness of interventions for people with disabilities in low‐ and middle‐income countries (Saran et al., 2020).

EGMs are becoming increasing popular as an approach to systematically identify, report and visualise the range of research activity in broad topic areas or policy domains. EGMs can provide a foundation for further, more focused research synthesis. They guide users to high‐quality research, inform research priority setting and help to define the focus of evidence synthesis such as systematic reviews. EGMs typically show what evidence is there, not what the evidence says, though there are exceptions.

However, one needs to be cautious of the application and use of these publications. There are various methods of mapping evidence (Saran and White 2018; Snilstveit 2014), which means all EGMs are not alike. A range of different approaches to evidence mapping and synthesis has been developed to support evidence‐informed policymaking. Each agency producing maps has a different approach, and it usually lies in one of the eight components of the EGM definitions: scope, type of evidence included, content of the map, structure of the map, transparency, visual or graphical display, map description and intended users.

For example, for nearly all the agencies, EGMs generally have a broad thematic scope covering a range of interventions and outcomes, except for the Evidence‐Based Policing Matrix (EBPM), which has just one outcome rather than set of outcomes. The EBPM is also an exception in being one of the maps that report what the evidence says (size, direction and significance of effects).

Approaches vary in terms of presentation of maps as well. Some use visualisation, like 3ie and Campbell, whereas others only present findings as descriptive reports such as the Global Evidence Mapping Initiative and the Collaboration of Environmental Evidence.

Campbell has a long‐standing track record of being one of the pioneers in synthesising and promoting the dissemination of high‐quality, unbiased evidence and helping our readership to understand why standards and principles in evidence‐making matters. This devotion goes into the setting of conduct and reporting standards for evidence mapping. Campbell EGMs include a visual presentation of the evidence as a matrix. Most usually, the matrix shows intervention categories (rows) and outcome domain (columns). The map may have additional dimensions capturing study or intervention characteristics, such as study design, location and population sub‐group, which can be applied as filters. The map is interactive: users click on entries to see a list of studies, and on study names to access a database record for the study. Campbell has produced and innovated in maps to a range of research questions.

Priority points to consider for EGMs are as follows.

1. They can be applied to a range of research questions such as of studies of effects, prevalence, implementation and barriers or facilitators.
2. The type of evidence included in the EGM depends on the research questions. A mega‐map (Saran et al., 2019) includes only systematic reviews and EGMs, while a typical effectiveness map will include systematic review and impact evaluations. There is even a “map of maps” that includes only other EGMs (Phillips et al., 2017).
3. Developing the framework, in particular the primary dimensions (row and column headings) is the most important—and often the most difficult—part of developing an evidence map.
4. The secondary dimensions may comprise study design, population, region, country and can be used as filters for relevant evidence.
5. Stakeholder consultation, particularly consultation with the funder(s), is an important stage in developing the map—most notably the framework.
6. The title of the EGMs should define the scope of the map.
7. EGMs have a broader scope than systematic reviews and the scope is defined by PICOS.
8. The reports are always accompanied by plain language summaries to facilitate research use.
9. It is desirable to have a maintenance plan to update maps annually.

Campbell has been working with different stakeholder agencies on innovations in evidence mapping. As already mentioned, we have worked on maps with different scopes: EGMs, mega‐maps and the map of maps. Whilst most maps to date are effectiveness maps, we have also produced maps of process evaluations for interventions for people experiencing homelessness, and one of tools, methods and metrics for studies analysing nutrition‐agriculture linkages. Country evaluation maps capture all evaluations of socioeconomic interventions in a single country—such maps are ongoing for Kenya, Uganda and the Indian state of Karnataka. There is also a map of just effectiveness studies for India.

As intended, maps are used in various ways. The child welfare mega‐map was used by UNICEF in its decision to fund an EGM of violence against children. The homelessness maps commissioned by the Centre for Homelessness Impact are being used as the basis for three systematic reviews. The Uganda Country Evaluation Map is being used in the planning process by the Office of the Prime Minister.

Do you have an interesting idea, topic or suggestions for an EGM? We would love to hear your thoughts. You can also register with us to join the innovation!
